# Identification of Factors Contributing to Traffic Accidents amongst Girls in Tehran with Specific Focus on Psychosocial Factors

**DOI:** 10.29252/beat-080104

**Published:** 2020-01

**Authors:** Siyamak Tahmasebi, Seyyed Mohammad Hossein Javadi, Tahereh Azari Arghun, Forughe Edrisi, Alireza Tajlili

**Affiliations:** 1 *Social Work Department, University of Social Welfare and Rehabilitation Sciences, Tehran, Iran *; 2 *Preschool Department, University of Social Welfare and Rehabilitation Sciences, Tehran, Iran*; 3 *Department of Psychology, University of Alzahra, Tehran, Iran*; 4 *Department of Psychology, Shahid Beheshti University of Medical Sciences, Tehran, Iran*; 5 *Country Coordinating Mechanism, Global Fund, Tehran, Iran*

**Keywords:** Psychosocial factors, Traffic accidents, Automobile driving, Young girls

## Abstract

**Objective:**

To identify the human factors contributing to traffic accidents with a special focus on psychosocial factors amongst young girls of Tehran, Iran.

**Methods:**

In a descriptive study conducted in Tehran, Iran in 2013, 108 girls aged 18-24 were enrolled by using a stratified cluster sampling method. Participants filled a wide range of validated questionnaires about traffic psychology.

**Results:**

The developed psychological model about behaviors of drivers’ factors as well as agreeable and aggressive personality trait with B coefficient of 0.25% and 0.37% were able to predict violation, driving style, perception of police laws, and off hook scheme and the mistrust with B coefficient of 0.33%, 0.23% and 0.28% in the level of 0.1 were able to predict violations and lapses of sample group, respectively. Extroversion with B coefficient of 0.27% also predicted unintentional violations of girls. B coefficient for perception of police laws was 0.22%. This was 0.25% for openness to experiences. Concerning driving accidents, the perception of police rules has the highest predictability.

**Conclusion:**

According to the results of the current research amongst girls in Tehran, a gender-sensitive interventional model can be designed for reduction of traffic accidents for this population group.

## Introduction

Traffic accidents and relevant problems and crimes are the problems which threat physical health and public welfare of population who are living in megacities. It is obvious that human factors are the most important factors of traffic accidents. Human factors not only are the main player in committing a violation, but also form and influence other factors such as vehicles, environment, and road. About 1.2 million people die annually because of traffic accidents all around the globe, while the number of injuries of traffic accidents may reach to 50 million [1]. 

It is estimated that without any practical innovation, the number of death and injuries will be increased by 65% during 2000-2020. This is predicted that during this period, the number of death and injuries because of traffic accidents in low and middle income countries will be increased more than 80% [1]. Researches in Iran revealed that mortality rate in the country was 30 per 100,000, while globally this figure was 22.6 per 100,000. The mortality rate due to traffic accidents constitutes 2.9% of total mortality at global level, while this percentage is 7.5 in Iran [2]. 

Furthermore, the ratio of Year of Life Lost (YLL) of traffic accidents because of traffic accidents is 2.5% and 13.5% in the world and in Iran, respectively. These findings clearly prove that the mortality rate of traffic accidents in Iran is higher than global and regional average. This ratio is not only higher in populations; but also, for any reason, higher than the total mortality and YLL rates and relative ratios in EMRO and worldwide. Numerous researches in traffic and transportation fields have revealed that human factor contributes to 85-90% of traffic accidents [2]. 

Most of these researches which have focused on relationship between human factors and traffic accidents revealed that high risk driving and violence during driving time are the predictors of most of the traffic accidents [3]. Research on personality of derivers has a significant importance, as well. Adventurist drivers are more susceptible to pass the stretch lines or do high risk maneuvers at the time of driving, which can threat themselves or other drivers. This kind of behavioral personality and fearless behaviors can lead to most of traffic accidents [4]. In addition, other researches have proved that personality specifications have some relationships with driving accidents [5]. 

Results of different researches about gender specification are variable. Waylen and McKenna concluded that traffic accidents in both genders are high; however, the models of traffic accidents are different in both genders [6]. Driving is a complicated psycho-motor function which needs a perfect harmonization between motor and sensory system of the body. This function can be affected by Emotional Motivation perception, learning, memory, notice, concentration, passion, time estimation, and visual, audio and decision making functions [7].

A great number of researches proved that social attitude and sociologic vision which can be emerged in law perception, attitude towards police, violence and delinquency, social satisfaction and national and religious identity may affect traffic accidents [7]. Furthermore, there are a number of researches revealed that there might be an accordance between lifestyle and driving behavior [8]. In this research, a number of models were applied to define the role of human factors in traffic accidents of young girls in Tehran, Iran with a focus on important psychosocial variables, including Shope and Bingham [9], Morrongiella and Lessard [10], and Parker [11] models.

This study was designed based on the findings from previous studies. Consequently, the research questions of this study were (i) What are the psychological factors associated with traffic accidents of girls aged 18-24 years? (ii) To what extent, these factors affect the traffic accidents in this age/sex group? (iii) What are the social factors associated with traffic accidents in this age/sex group?

## Materials and Methods

*Research design*

This quantitative descriptive study was conducted in Tehran, Iran in 2013. This study gathered information about a wide range of social and psychological factors that may contribute into traffic accidents. 

*Population and sampling*

The participants of the study were girls aged 18-24 years who were living in Tehran, Iran with an experience of traffic accidents and their accidents have been registered in one of the relevant organizations including hospitals (because of injury or death), insurance organizations (because of damages) or judiciary system (i.e., correctional centers or jails). The applied method for sampling was stratified sampling method. 

*Exclusion criteria*

As the study was self-reported, minimum education for understanding and filling the questionnaires was mandatory. Cases with mental disorders, physical limitations or low education level who were not able to fill the questionnaires were excluded from the study.

*Measuring tools*

Data collection tools in this research were questionnaires including (i) demographic questionnaire*.* In this questionnaire, the characteristics such as age, gender, occupation, level of education, time of obtaining driving license, past history of traffic accidents and their time of occurrence, car or vehicle type, accident place and other relevant factors to traffic accidents including wearing a seatbelt, talking with cell phone and driving under effect of alcohol and drugs were explored. Attitude of respondents about traffic accidents was another topic included in the questionnaires. Three types of questions were in the questionnaires such as questions with Likert scales, binary questions and open-ended questions. 

(ii) General health questionnaire that was originally designed by Goldberg and Hiller [12]. This was validated for Iranian population by Taghavi *et al*. In this study, test-retest reliability of this questionnaire was 0.7 and half reliability of the questionnaire was 0.93. Alpha Cronbach was also calculated for this study which was 0.9 [13]. (iii) Lifestyle questionnaire that was designed by Alhamoud, Simonzo El Sphar merging a wide range of topics from different individual and socio-cultural factors. The economic factors were also added in this questionnaire as well as other traffic related factors such as stress, road, environment, and past history of the drivers. The research team reported an acceptable expert validity for this questionnaire. The Alpha Cronbach for this questionnaire was 0.7. [8]

(iv) Manchester driving behavior questionnaire [MDBQ] that was designed by Reason *et al*. in Manchester University [14]. The main concept of this questionnaire was separation of mistakes and violations to law. The researcher believed that psychological causes of these outcomes were different, thus needed to be treated differently. This questionnaire was also tested from reliability point of view by Arizi and Haghayegh. They found out that lapses, mistakes and violations [intentional or unintentional] needed to be classified and approached differently [15]. They also found out that every group of factors had high internal consistency coefficients. Compared to the total score, the internal consistency coefficient was 0.77 for laps, 0.81 for mistakes, 0.86 for intentional violations and finally 0.65 for unintentional violations. 

**Table 1:** Descriptive demographic findings

**Variable**	**Percent**	**Frequency**
Sex [N=108]		
Female	100	108
Marriage Status [N=108]		
Single	76.8	83
Married	23.2	25
Age [N=108]		
18-19	11.11	12
20-21	28.7	31
22-23	33.33	36
24-25	26.85	29
Education [N=108]		
Less than high school	1.85	2
High school	12.03	13
University student	61.1	66
University graduate	25	27
Father’s education [N= 108]		
Illiterate	3.7	4
8^th^ grade or less	23.14	25
High school graduate	43.51	47
Bachelor	22.22	24
Master or higher	7.4	8
Mother’s education [N=108]		
Illiterate	10.18	11
8^th^ grade or less	28.7	31
High school graduate	49.07	53
Bachelor	7.4	8
Master or higher	4.62	5
Driving license [N=108]		
No	15.74	17
Yes	84.25	91
Driving year experience [N= 108]		
1-2 Years	50	54
3-4 Years	26.85	29
5-6 Years	19.44	21
7-8 Years	2.77	3
9-10 Years	0.92	1
Accident experience [N=108]		
1 time	46.29	50
2 times	28.7	31
3 times	16.66	18
4 times	7.4	8
5 times or more	0.92	1

**Table 2:** Regression Parameters

**P value**	**T**	**Standardized coefficients**	**Non-standardized coefficients**		**Model**
			**Standard Error**	**B Coefficient**	
**Behavioral Variables Model**					
0.0001	9.769		0.181	1.765	fixed
0.001	3.279	0.192	0.221	0.724	Use of drug and alcohol
0.13	-2.50	-0.147	0.161	-0.404	Driving’s license
0.0001	5.198	0.303	0.30	0.156	Driving age
**Environmental variables model**					
0.0001	11.911		0.138	1.644	Fixed
0.018	2.406	0.228	0.181	0.435	Road

**Predictor**: Driving age, Use of drug and alcohol, Driving’s license, Road. **Dependent variable**: Accident record.

**Table 3:** Regression Parameters

**P value**	**T**	**Standardized coefficients**	**Non-standardized coefficients**		**Model**	
			**Standard Error**	**B Coefficient**		
**Accidents Model**						
0.007	2.749		0.562	1.545	Fixed	
0.007	2.744	0.252	0.29	0.79	Perception of police rules	
0.15	-2.46	-0.226	0.22	-0.54	Openness to experience	
**Intentional violations model**						
					
0.65	1.863		6.191	11.533	Fixed	
0.0001	4.437	0.354	0.336	1.489	Aggression	
0.002	3.114	0.254	0.214	0.665	Agreeableness	
0.15	2.475	0.183	0.263	0.651	Perception of police rules	
0.19	-2.38	-0.176	0.323	-0.771	Extroversion	
0.29	2.214	0.179	0.165	0.365	Mistrust [father]	
**Mistakes model**						
0.150	-1.45		14.226	-20.626	fixed	
0.003	3.099	0.276	0.208	0.645	Abandonment [mother]	
0.005	2.870	0.233	0.748	2.146	Family	
0.009	2.650	0.231	0.385	1.020	Aggression	
0.15	2.475	0.202	0.305	0.754	Perception of police rules	
**Lapses model**						
0.0001	5.070		4.401	22.312	Fixed	
0.002	3.248	0.250	0. 86	0.280	Mistrust [mother]	
0.0001	-4.90	-0.348	0.114	-0.558	Driving style	
0.0001	4.207	0.302	0.140	0.588	Perception of police rules	
0.006	2.793	0.214	0.110	0.308	Agreeableness	
0.19	-2.38	-0.176	0.153	0.364	Regional nation dignity	
**Unintentional Violations Model**						
0.255	-1.14		2.973	-3.400	Fixed	
0.005	2.861	0.263	0.156	0.446	Family	
0.25	2.277	0.209	0.74	0.170	Aggression	

**Predictor**: Perception of police rules, openness to experience, Aggression, Agreeableness, Extroversion, Mistrust[father], Abandonment [mother], Family, Mistrust [mother], Driving style, Regional nation dignity. **Dependent variable**: Accident record.

**Table 4:** Fitness indicators for assessment of driving behaviors models

** Indicator**	**Absolute Indicators**						**Comparing Indicators**			
**Model**										
	**K** ^2^	**P**	**df**	**K** ^2^ **/df**	**GFI**	**AGFI**	**NFI**	**TLI**	**CFI**	**RMSEA**
Intentional violation [Female]	3695.95	0.00	824	4.48	0.70	0.64	0.58	0.57	0.63	0.09
Mistakes [Female]	1890.73	0.00	956	1.97	0.83	0.80	0.86	0.91	0.92	0.05
Lapses [Female]	2220.02	0.00	584	3.80	0.74	0.69	0.61	0.61	0.66	0.09

(v) Young parenting inventory questionnaire that was originally designed by Young for classifying childhood schema [16]. Salavati tested all the 72 items of this inventory in Iran and the results of reliability coefficients were 0.6 and 0.8 for mothers and fathers, respectively [17]. (vi) NEO-five factor inventory [NEO-FFI] questionnaire that is a well-known and widely used tool for identification of personality traits. It classifies them into five major personality groups [i.e. extraversion, conscientiousness, openness to experience, neuroticism and agreeableness]. The independence of these major five groups was confirmed by Salavai. He applied a factor analysis in his test on students studying in Shiraz University, Shiraz, Iran [18]. Alpha Cronbach was 0.88 for neuroticism and 0.77 for extraversion. In the current study, reliability and validity of all questionnaires were tested and confirmed. 

*Method of conducting the study*

In order to gather the data, a number of active volunteers of youth organization of Red Crescent Society who had a relevant background were selected after an interview and then trained for filling the questionnaires. Their level of readiness was tested through another interview. There was also another monitoring mechanism in place in order to guarantee the quality of data gathering process. 

*Method of data analysis*

Mean and standard deviations were used at the first step for comparing the variables. Correlation of the variables was also checked. All these analysis plus simultaneous regression, step-wise regression and structural equation modeling were conducted by SPSS software (Vesion 20, Chicago, IL, USA) and Lisrel software. For the purpose of modeling, by LISREL 8.70, instructive equations at latent variable level were applied. In order to check the reliability and validity of research tools, Confirmatory Factor Analysis was done. 

This was followed by reviewing of relations between latent variables and model fitness. Maximum Likelihood Analysis was considered in all the stages. For assessing the fitness of measurement, Goodness of Fit Index, Akaic Information Criterion, K2 index, K2/df, Comparative Fit Index, Normed Fit Index, Non-normed Fit Index, Root Mean Square Error of Approximation, Standard RMR, and SRMR were applied.

*Ethical principles*

Ethics committee of University of Social Welfare and Rehabilitation reviewed all the research documents and after thorough review of them approved them on 16^th^ July, 2013 (Reg. USWR.REC.1393.104). 

## Results

*Descriptive demographic findings*

All cases were female, out of which 76% were single and 23% were married. Furthermore, 11% of the participants aged 18 to 19 years, 28% were 20 to 21 years, 33% aged 22 to 23 years, and about 26% were 24 to 25 years old. Regarding educational status, 12% of cases were studying at or had finished their high school, 61% were university students and 25% had graduated from university. The findings were summarized in the Table 1.

*Findings of stepwise regression analysis for behavioral and environmental variables*

All environmental factors [such as required time for obtaining driving license, history of accident, wearing seatbelt, alcohol and drug use, focus and concentration, road and satisfaction with driving lessons] were included into the simultaneous regression equation as predicting factors. The findings were summarized in Table 2. As per findings, from environmental factors category, road can predict 0.04 of changes in the accident history and as per observed F [5.78], it was statistically significant [α=0.01]. Results of Ultimate Regression Analysis for Accidents Model, psychosocial factors model affecting intentional violations model, Mistakes Model, Lapses Model & Unintentional Violations Model were demonstrated in Table 3.

*Model of fitness and comparing the indicators among cases*

After development of structural equation on the basis of theoretical model in LISREL software, fitness indicators for primary model were assessed. As it can be seen in Table 4, although the fitness of this model can be categorized as acceptable, the degrees of freedom of this model were high. In Table 4, the fitness indicators were summarized. These indicators are calculated through analysis of revised model. Following applied approaches for assessment of the fitness of the models; (i) Chi-square test revealed that it was not significant, and the model was considered at desirable level of fitness. 

(ii) Root Mean Square Error of Approximation [RMSEA] denoted to less than 0.05 desirable and between 0.05-0.08 acceptable fitness. (iii) Goodness of Fit Indicators [GFI] displayed adjusted goodness of fit indicators, normed and non-normed fitness indicators [NFI and NNFI] indicating more than 0.9 desirable fitness. (iv) SRMR illustrated less than 0.05 desirable fitness. Putting all this factors together, two factors had a better fitness model, namely intentional violation and laps. The latter was just true for boys. This fitness also needed to be enhanced by more modifications. 

## Discussion

In order to check the role of different contributing factors to accidents and behaviors at the time of driving, different factors about personality, mental health and other psychological factors including self-esteem and aggression were included in multiple regression analysis. All factors with desirable predictability for accidents and behaviors at the time of driving, were included in a structural equation model in order to test the fitness. Results of stepwise regression regarding driving accidents at final step showed that two factors of perception of police laws and openness to experiences could predict accidents among girls with B coefficient of 22% and 25%, respectively. So perception of police laws factor was the strongest predictor of accidents. Correlation results of accidents record with lifestyle aspects of girls sample demonstrated only significant correlation between perception of police rules factor and accident record [*p*<0.01, R=25%]

The findings were consistent with previous findings [20] revealing that perception of police rules with B coefficient of 82% was the most important predictor of violation and accident. The other compared factors were life stress, perception of police commands, social factors and driving style. Javadi *et al*. also reported that perception of police commands was the most important predictor of traffic accidents among boys which was in line with the findings of this research [16]. 

The present study indicated that only personality trait of openness to experience can negatively predict accidents among girls. The more experience, the less the rate of lapses, mistakes and violations. Openness to experience points to this characteristics were active imagination, aesthetic sense, attention to internal emotion, mental curiosity and judgment independence. This type of people was curious about both internal needs and external world and their life was full of experience. 

The individuals with lower score were willing to have common behavior in this area, resisted changes and their emotional answers were very limited. This type of people was narrow minded and had superficial and objective points of view of the world around them. It seems that connection of this characteristic was contrary to accidents, and this group had superficial and objective mind and emotional answers in different situations, the less interest to get new experience, the more difficult to be in new situation and it was possible not be able to manage the situation well. 

The results reported by others indicated to a significant difference between open to experience factor of risky and normal drivers. So the mean of openness to experience of high risky drivers were lower than normal ones. People who applied valuable system with strong conscience together in various situations got higher score of openness to experiences’ index, so that from view point of psychologists, openness to experience index was equal or healthier. These people with such characteristics drove safely. 

Generally, perception of police laws and personality trait of openness to experience can predict accidents better. It seems that perception of police regulation factor was stronger predictor than openness to experience among girls which showed that attitudes toward rules and individual perception about traffic limitation of sample group were very important for reducing accidents rate. 

Based on previous reports for boys, some youth believed that rules and regulation were not effective and deterrent enough or were not enforced or sometimes could be bypassed. Therefore, updating the rules more frequently and in shorter intervals based on the field assessments is recommended. These rules are needed to be trained sufficiently at community level. Finally, this is very important to begin the trainings about caution, following the rules and discipline from the younger ages and during school times. This can be complemented in universities in continuous and unified approaches [20].

The results of research about driving aspects indicated that agreeable and aggressive personality traits with B coefficient of 0.37, 0.25 could predict violations among girls. Moreover, driving style, perception of police laws and scheme of freedom and mistrust with B coefficient of 28%, 0.23%, 0.33% in level of 0.01 could also predict violations and lapses of sample group, respectively. Extroversion with B coefficient of 0.27 was also unintentional violations predictor of girls; while aggressive factor was the strongest predictor of intentional violations and driving style factor was the strongest predictor of lapses and mistakes.

Therefore, considering the available items of driving style in the questionnaire, the factors which affected occurrence of accidents and driving violations and mistakes more than other factors were related to using cell phone, fastening seat belt, eating and drinking during driving, allocated time to arrive destination, purpose of driving, and way of driving training. There was contrary connection between extroversion and unintentional violations of girls. It means that the more extroversion score of individuals, the less possibility occurrence of all kinds of unintentional violations and mistakes. 

These findings are not consistent with research results done before, but it is explanatory for the purpose of intentional or unintentional violations, due to the tendency of people toward intentional violations in order to satisfy their needs of motivations. If the extroversion is less, the possibility of intentional violations is less and unintentional violations are more. Agreeable personal traits include interpersonal willing area, having tendency toward humanism, sympathy with others, and enthusiasm for helping people and belief in other’s help mutually.

People get lower scores are ones who are quarrelsome, egotism, having doubt about others, and they are more competitive instead of cooperative. The lower score of this field happened together with egotism, antisocial, and paranoia. While higher score of this field has relationship with dependence modes, the findings of present research is consistent with previous ones reporting that extroversive and agreeable personality trait of drivers are contrary to rate of mistakes and illegal action. It means that the more agreeable and extroversive personal traits, the less possibility of conducting mistakes and illegal action of driving [21].

From another point of view, some of the important factors from category of social factors such as carrying out rules, experience of driving, road status and satisfaction with the driving training institute could predict the accidents among girls. The record of imprisonment and crime, weather condition, concentration and driving age could predict aspects in driving behavior. The more getting driving experience, the less risky rate of beginner drivers; the risky rate of getting involved in accident to be guilty has relatively decreased by 6% in return for every year after getting the driving license [21]. 

The decreasing rate happened more among female drivers. Individuals with more driving experience commit less traffic violations than beginners. Meanwhile, it is likely that younger people have risk of personality factors as excitement follows risk in consistent with a previous research [21].Lack of concentration can also negatively predict accidents, lapses, mistakes and violations among girls in our study. It means that more concentration on driving led to less occurrence of accidents, mistakes, violations and vice versa.

Any factor which distracts the driver from the main task of driving is categorized under neglection. This factor plays an important role in traffic accidents, especially novice drivers. These drivers may underestimate the importance of road situation including the quality of pavements or frozen and slippery roads. Neglecting and similar factors are mostly underreported by police; however, according to even these reports, 32% of 16-year-old individual’s accidents were due to neglecting. 

It is more likely that younger known guilty because of neglecting have less experience about multi-dimensional factors such as information processing, understanding, and applying of behavioral driving task. In addition to concentration [negatively], driving age, record of imprisonment and crime, computer games and fine were able to predict violations among social factors affecting on intentional violations of female group. Cultural effects, role of video, films and the role of car’s advertisements on risky driver’s behavior has been determined, while personal interests to motorcycling race also falls in this category. It seems that computer games related to driving and motorcycling could affect traffic violations through observational trainings among girls.

The findings of this study confirmed that a complex of different factors contribute to the traffic accidents and behaviors of drivers such as driving under the influence of drugs and alcohol that can lead to traffic accidents, or people who had a history of crime or detention had a higher probability of car accidents. This study tried to highlight the effect of psychological factors in traffic accidents. The prevalence of these problems is growing in the societies and cases with aggression or depression symptoms. 

These factors can affect the behaviors of drivers and can be worsened, if lack of public knowledge is added to this challenge. Accumulation of these challenges can synergistically lead to social consequences. Thus, this is necessary to develop some multidimensional initiatives to properly address this challenge. This research tried to develop a wide range of models in order to explore different aspects of psychological factors in traffic accidents. This approach led to some sub scales. 

To have a significant finding in these subscales, more cases were required. This is one of the weaknesses of this research. Research team believes that non-significant findings in some areas including self-reliance and attention can be the result of this shortage. The absence of reliable and distinct base for getting statistical information related to accidents should also be mentioned. The lack of cooperation of some hospitals should be considered and the little sample volume in some analysis for entering variables simultaneously must be noted too.

## References

[1] Moskal A, Martin JL, Laumon B (2012). Risk factors for injury accidents among moped and motorcycle riders. Accid Anal Prev..

[2] Waylen A, McKenna F (2002). Cradle Attitudes–Grave Consequences: The Development of Gender Differences in Risky Attitudes and Behaviour in Road Use: Summary Report.

[3] Salavati M (2007). Most schemas and schematherapy efficacy in patients with borderline personality disorder. Clinical Psychology, phD Thesis.

[4] Andersson AL, Bunketorp O, Allebeck P (1997). High rates of psychosocial complications after road traffic injuries. Injury..

[5] Shope JT (2006). Influences on youthful driving behavior and their potential for guiding interventions to reduce crashes. Inj Prev..

[6] Amoros E, Martin JL, Laumon B (2006). Under-reporting of road crash casualties in France. Accid Anal Prev..

[7] Violence WHO (2013). Prevention I, Organization WH. Global status report on road safety 2013: supporting a decade of action.

[8] Al-Hemoud AM, Simmons RJ, Al-Asfoor MM (2010). Behavior and lifestyle characteristics of male Kuwaiti drivers. J Safety Res..

[9] Shope JT, Bingham CR (2008). Teen driving: motor-vehicle crashes and factors that contribute. Am J Prev Med..

[10] Morrongiello BA, Lasenby-Lessard J (2007). Psychological determinants of risk taking by children: an integrative model and implications for interventions. Inj Prev..

[11] Parker D, Lajunen T, Summala H (2002). Anger and aggression among drivers in three European countries. Accid Anal Prev..

[12] Korchagin V, Ljapin S, Rizaeva J, Konovalova V (2017). Subsystem of road accident consequences elimination methodology of subsystem efficiency improvement. Transportation Research Procedia..

[13] Organization WH (2015). Global status report on road safety 2015.

[14] Arizi H, Haghayegh A (2009). Psychometrics affections of Manchester Driving Behavior Questionnaire. Payesh..

[15] Javadi SMH, Azad HF, Tahmasebi S, Rafiei H, Rahgozar M, Tajlili A (2015). Study of psycho-social factors affecting traffic accidents among young boys in Tehran. Iranian Red Crescent Medical Journal..

[16] Twisk DA, Vlakveld WP, Commandeur JJ, Shope JT, Kok G (2014). Five road safety education programmes for young adolescent pedestrians and cyclists: a multi-programme evaluation in a field setting. Accid Anal Prev..

[17] Shope JT (2006). Influences on youthful driving behavior and their potential for guiding interventions to reduce crashes. Inj Prev..

[18] Taghavi SMR (2001). Assessing reliability and validity of the General Health Questionnaire (GHQ). Persian Journal of Psychology..

[19] Al-Hemoud AM, Simmons RJ, Al-Asfoor MM (2010). Behavior and lifestyle characteristics of male Kuwaiti drivers. J Safety Res..

[20] Javadi S-M-H, Tahmasebi S, Azari-Arghun T, Arshi M, Alipour F (2017). The youth and experience of traffic accidents (Grounded theory). Disaster Health Quarterly..

[21] Zuckerman M, Neeb M (1980). Demographic influences in sensation seeking and expressions of sensation seeking in religion, smoking and driving habits. Personality and individual Differences..

